# An m6A/m5C/m1A/m7G-Related Long Non-coding RNA Signature to Predict Prognosis and Immune Features of Glioma

**DOI:** 10.3389/fgene.2022.903117

**Published:** 2022-05-26

**Authors:** Dongqi Shao, Yu Li, Junyong Wu, Binbin Zhang, Shan Xie, Xialin Zheng, Zhiquan Jiang

**Affiliations:** Department of Neurosurgery, The First Affiliated Hospital of Bengbu Medical College, Bengbu, China

**Keywords:** M6A, m5C, m1B, m7G, glioma, RNA methylation, long non-coding RNA, prognostic signature

## Abstract

**Background:** Gliomas are the most common and fatal malignant type of tumor of the central nervous system. RNA post-transcriptional modifications, as a frontier and hotspot in the field of epigenetics, have attracted increased attention in recent years. Among such modifications, methylation is most abundant, and encompasses N6-methyladenosine (m6A), 5-methylcytosine (m5C), N1 methyladenosine (m1A), and 7-methylguanosine (m7G) methylation.

**Methods:** RNA-sequencing data from healthy tissue and low-grade glioma samples were downloaded from of The Cancer Genome Atlas database along with clinical information and mutation data from glioblastoma tumor samples. Forty-nine m6A/m5C/m1A/m7G-related genes were identified and an m6A/m5C/m1A/m7G-lncRNA signature of co-expressed long non-coding RNAs selected. Least absolute shrinkage and selection operator Cox regression analysis was used to identify 12 m6A/m5C/m1A/m7G-related lncRNAs associated with the prognostic characteristics of glioma and their correlation with immune function and drug sensitivity analyzed. Furthermore, the Chinese Glioma Genome Atlas dataset was used for model validation.

**Results:** A total of 12 m6A/m5C/m1A/m7G-related genes (AL080276.2, AC092111.1, SOX21-AS1, DNAJC9-AS1, AC025171.1, AL356019.2, AC017104.1, AC099850.3, UNC5B-AS1, AC006064.2, AC010319.4, and AC016822.1) were used to construct a survival and prognosis model, which had good independent prediction ability for patients with glioma. Patients were divided into low and high m6A/m5C/m1A/m7G-LS groups, the latter of which had poor prognosis. In addition, the m6A/m5C/m1A/m7G-LS enabled improved interpretation of the results of enrichment analysis, as well as informing immunotherapy response and drug sensitivity of patients with glioma in different subgroups.

**Conclusion:** In this study we constructed an m6A/m5C/m1A/m7G-LS and established a nomogram model, which can accurately predict the prognosis of patients with glioma and provides direction toward promising immunotherapy strategies for the future.

## 1 Introduction

Gliomas, including both low-grade gliomas (LGGs) and glioblastomas (GBMs), are the most common and fatal type of malignant tumor of the central nervous system (Wang et al., 2020c). According to the standards formulated by the World Health Organization, LGGs are defined as lower-stage gliomas (I–III) and are associated with good prognosis. In contrast, GBMs are classified as the highest grade of glioma (IV) and patients have worse prognosis (Aldape et al., 2015; Gittleman et al., 2020). At present, the main treatment for gliomas is surgery combined with postoperative radiotherapy and chemotherapy. However, although scientists have achieved some progress and improvements in both clinical and basic research, as well as comprehensive treatment, of gliomas in recent years, the overall curative effect for gliomas, particularly GBMs, remains poor, and the prognosis of affected patients is very poor. Approximately 88% of patients with GBM have a 5-years survival rate of <5%, and the overall median survival time is only around 14 months (Tykocki and Eltayeb 2018). The main treatment strategy also has a number of disadvantages (Martínez Bedoya et al., 2021; Liu Z. et al., 2022). For example, due to the complexity of, and dynamic changes in, the tumor microenvironment, patients frequently exhibit resistance to chemotherapeutic drugs. Further, the low selectivity of radiotherapy and chemotherapy causes injury to normal brain tissues, and the use of chemotherapeutic drugs can lead to systemic immunosuppression (Cui et al., 2018; Jackson et al., 2019; Dumas et al., 2020; Minniti et al., 2021). Therefore, identification of more effective treatment methods is needed to improve the outcomes for patients with GBM.

Epigenetic changes, including DNA methylation, histone covalent modification, chromatin remodeling, non-coding RNA (ncRNA) activity, and RNA chemical modification, are commonly associated with various types of tumorigenesis, malignancy, and therapeutic resistance (Wang E. et al., 2021; Wu et al., 2022). Further, there is increasing evidence that dynamic RNA modification pathways are mis-regulated in human cancers, including gynecological cancers, bladder cancer, and GBM, among others, and may be an ideal target for cancer therapy (Chai et al., 2019; Liu 2021; Huang et al., 2022). RNA post-transcriptional modifications, as a frontier and hotspot in the field of epigenetics, have become a considerable research focus in recent years. Among the known RNA modifications, methylation is most abundant, including N6-methyladenosine (m6A), 5-methylcytosine (m5C), N1 methyladenosine (m1A), and 7-methylguanosine m7G (Zhou et al., 2021) methylation. Of these, m6A is the most abundant form of methylation modification in eukaryotic RNA and the most thoroughly studied type of RNA modification. Sequences flanking m6A modification sites in messenger RNA (mRNA) are highly conserved, and this modification mainly occurs in RRACH (where R = purine, A = m6A, and H = non-guanine) motifs on the adenine. The function of m6A modification is determined by methyltransferases (writers: e.g., methyltransferase-like 14, WT1-related protein, and methyltransferase-like 3) and demethylases (erasers: e.g., fat mass and obesity-related protein and ALKB5 homologs [ALKBH5, etc.]; and readers: e.g., Yth-domain–containing family, insulin-like growth factor 2 mRNA–binding protein family, and heterogeneous nuclear ribonucleoprotein A2/B1) (Visvanathan et al., 2018; Zhang Y. et al., 2020). m5C modification of RNA is widespread in cells and has important roles in regulating gene expression and RNA stability. In addition, m5C methylation is closely related to proto-oncogene activation, and the m5C-modified methyltransferase, NSUN2, is differentially expressed in tumor and para-cancer tissues (Dong and Cui, 2020). m1A modification affects the first nitrogen atom of the adenine base and carries a positive charge under physiological conditions. Moreover, m1A modification affects the tertiary structures of ribosomes and gene translation, with important functions in regulating gene expression and controlling cell fate, and thus affects disease occurrence and development (Alriquet et al., 2021; Zheng et al., 2021). Research suggests that m7G is present in eukaryotic mRNA 5′ caps and at defined internal positions within transfer RNA (tRNA) molecules and ribosomal RNAs across all domains of life. Typical enzymes involved in regulating internal m7G methylation include methyltransferase-like 1 (the YEAST enzyme homolog of TRMT8) and its co-factor, WDR4, which catalyzes m7G modification at G46 of specific tRNAs, such as tRNAPhe (Pandolfini et al., 2019).

Long ncRNAs (lncRNAs) are a class of RNAs of >200 nucleotides that do not encode proteins. In cancer, lncRNAs are important epigenetic regulatory molecules, and their abnormal expression can constitute an effective biomarker for use in early diagnosis and monitoring the effects of treatment (Bhan et al., 2017; Peng et al., 2017). However, there is currently no evidence to explain the association between gliomas and the lncRNAs associated with four major RNA-methylation modification. In this study, we extracted expression data for 49 m6A/m5C/m1A/m7G genes from The Cancer Genome Atlas (TCGA) database, then used bioinformatics and statistical analysis methods to explore the relationships between lncRNAs associated with m6A/m5C/m1A/m7G and glioma diagnosis and prognosis. Furthermore, the Chinese Glioma Genome Atlas (CGGA) dataset was used for model validation.Subsequently, we analyzed the functions of the lncRNAs and their relationships with the immune microenvironment. Notably, we identified potential drugs that can target these lncRNAs, providing a potential novel direction for the treatment of gliomas.

## 2 Methods

### 2.1 Glioma Patient Database

First TCGA data portal was accessed to download and collate GBM and LGG gene expression profiles (https://portal.gdc.cancer.gov/) (Blum et al., 2018), along with relevant glioma patient clinical information, including age, sex, time of survival, survival, and availability of tissue or organ samples, which includes five normal and 698 tumor samples, Patients with primary tumor expression and survival information were included in this study. A list of 49 m6A/m5C/m1A/m7G genes was generated (Table 1), based on existing research (Cao et al., 2021; Wiener and Schwartz 2021). Gene expression profiles were then fully annotated using the Gencode project and mRNA and lncRNA profiles separated (Frankish et al., 2019). Pearson’s correlation analysis was used to screen m6A/m5C/m1A/m7G-related lncRNAs, and 621 m6A/m5C/m1A/m7G-related lncRNAs were identified using the threshold criteria, correlation coefficient |R | > 0.4 and *p* < 0.001. the data of prognostic prediction performance was download from the Chinese Glioma Genome Atlas (CGGA,http://www.cgga.org.cn) databases, including CGGA-325, and CGGA-693 (1018 glioma patients). The downloaded data includes clinical data and mRNA data. All the datasets from two datasets are normalized to fragment per kilobase million (FPKM) values. Patients without follow-up data or overall survival <30 days were excluded.

**TABLE 1:** m6A/m5C/m1A/m7G RNA methylation–related genes.

Gene	Coef
AL080276.2	-2.09085061330427
AC092111.1	-0.310248518121221
SOX21-AS1	-0.28683274752078
DNAJC9-AS1	-0.928895736570429
AC025171.1	0.940560063064456
AL356019.2	-1.39389951438732
AC017104.1	0.57019326721837
AC099850.3	0.288163704315659
UNC5B-AS1	1.74511337797007
AC006064.2	0.899857252034493
AC010319.4	0.442850890823364
AC016822.1	-1.04558931248538

### 2.2 Construction of a Predictive Signature

The complete TCGA dataset was randomly divided into a discovery cohort and a testing cohort. In the discovery cohort, prognostic m6A/m5C/m1A/m7G-related lncRNAs were first identified by univariate analysis (*p* < 0.05), followed by application of the least absolute shrinkage and selection operator (LASSO) method (McEligot et al., 2020), which showed that 12 lncRNAs associated with m6A/m5C/m1A/m7G were also associated with various glioma characteristics. LASSO is a linear regression methodology based on L1-regularization, where L1-regularization reduces model complexity and risk of overfitting. The foremost advantage of LASSO lies in the application of penalty multivariate analysis of all variable coefficients, as well as designation of comparatively unimportant experimental variable coefficients as zero, excluding them from the model (Wang et al., 2022; Yan et al., 2022). The selected 12 m6A/m5C/m1A/m7G-related lncRNAs were analyzed by multi-factor Cox regression to develop an m6A/m5C/m1A/m7G-lncRNA signature (LS), which was calculated as follows (Yue et al., 2021):

m6A/m5C/m1A/m7G-LS risk factor=∑exp(m6A/m5C/m1A/m7G-LncRNAs)×the coefficient of each m6A/m5C/m1A/m7G lncRNA from Cox analysis 

Low- and high-risk subgroups were then generated based on the median risk score. Whole-genome expression profiles, 49 m6A/m5C/m1A/m7G genes, 12 m6A/m5C/m1A/m7G lncRNAs, and the m6A/m5C/m1A/m7G-LS were analyzed by principal component analysis (PCA) to achieve model recognition (Xu et al., 2019), using the R package, “scatterplot3d”.

To explore potential differences between high and low-score groups (Ni et al., 2021), the Kaplan–Meier survival method was used to determine differences in clinical outcomes between the two groups (Shen et al., 2020). The LS and other factors (age, sex, risk score, and stage) were used to establish a predictive nomogram. Moreover, the Hosmer–Lemeshow test was applied to detect the goodness-of-fit of the nomogram (Lee and Jung 2019). The results of decision curve analysis (DCA) were plotted to quantify and assess the clinical value of the nomogram by the R package “ggDCA” (Sheng et al., 2022); DCA can be performed to obtain the clinical net benefit of the nomogram, compared with all or none of the strategies (Zhang et al., 2021).

Survival analysis, AUC values and multivariate analyses were performed on CGGA data to validate the prognostic prediction performance of the prognostic model in Chinese glioma patients (Ren et al., 2022).

### 2.3 Clustering of Samples Based on m6A/m5C/m1A-Related lncRNAs

The R software package “ConsensusClusterPlus” was used to divide tumor samples into different groups based on expression levels of the 12 prognostic lncRNAs in tumor samples and analyze their associations with prognosis through cluster analysis of samples in the database (Wilkerson and Hayes 2010). The R software package “limma” was used to filter differences in immune cell infiltration among different types of samples and m6A/m5C/m1A/m7G-LS group (Diehl et al., 2020). In addition, differences in the expression levels of programmed death-ligand 1 (PDL1) in various tumor subtypes were analyzed.

### 2.4 Gene Ontology (GO) and Kyoto Encyclopedia of Genes and Genomes (KEGG) Enrichment Analysis

GO and KEGG pathways were used for enrichment analyses (Ding and Zhang 2017) in the R package “clusterProfiler” (Yu et al., 2012); *p* < 0.05 was the threshold for significant enrichment of functional pathways.

### 2.5 Comparison of Tumor-Infiltrating Immune Cell Subpopulations Between Risk Groups

The CIBERSORT(Kang et al., 2022) algorithm was employed for immune infiltration estimation, to assess differences in immune cell subpopulations between high- and low-risk patients. Differences in immune cell landscape between the two risk groups were assessed using the Wilcoxon signed rank-sum test.

### 2.6 Immunotherapy Response Prediction

The R package “maftools” was used to analyze mutation data. Tumor-specific gene mutations were used to calculate the tumor mutation burden (TMB) (Xu et al., 2021). Using these data, combined with patient survival information, all samples were divided into high- and low-TMB groups, and the survival of patients in each group analyzed. Then survival rates were analyzed in four subgroups (low TMB + low risk score, low TMB + high risk, high TMB + low risk, and high TMB + high risk), to assess the relationship between risk score and patient survival rates (Lv et al., 2020). The tumor immune dysfunction and exclusion (TIDE) algorithm was used to estimate the probability of response to immunotherapy (Wang Q. et al., 2020).

### 2.7 Drug Sensitivity

To evaluate the relationship between drug sensitivity and the m6A/m5C/m1A/m7G-LS, half-maximal inhibitory concentration (IC_50_) values were assessed, as a reflection of the chemotherapeutic drug response. Using the R package “pRRophetic” (Wang et al., 2021d), the IC_50_ of different drugs was predicted for glioma samples, according to the Genomics of Drug Sensitivity in Cancer online tool.

## 3 Results

### 3.1 m6A/m5C/m1A/m7G-Related LncRNAs

**FIGURE 1:**
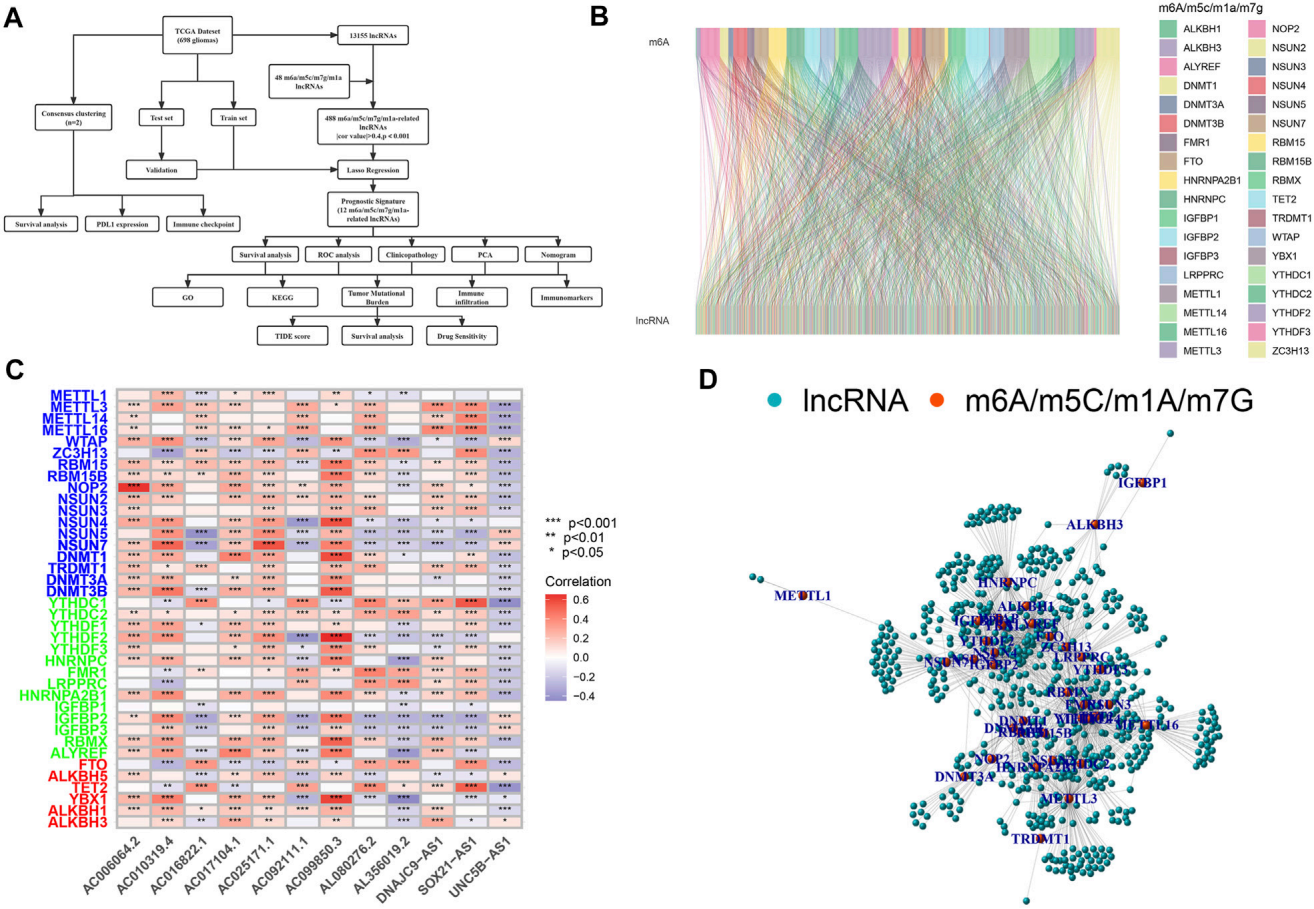
Selection of m6A/m5C/m1A/m7G-related lncRNAs in patients with glioma. **(A)** Flow chart of the study design. **(B)** Sankey diagram for the network of m6A/m5C/m1A/m7G genes and related lncRNAs. **(C)** Heatmap for relationships between m6A/m5C/m1A/m7G-related genes and lncRNAs. *p < 0.05, **p < 0.01, and ***p < 0.001. **(D)** lncRNAs co-expressed with m6A/m5C/m1A/m7G-related genes. Green, lncRNAs; red, m6A/m5C/m1A/m7G-related genes.

A schematic illustration of the construction of the m6A/m5C/m1A/m7G-related LncRNAs prognostic signature and subsequent analyses is presented in Figure 1A. A total of 48 m6A/m5C/m1A/m7G genes and 13,155 lncRNAs were extracted from the GBM and LGG datasets. We defined m6A/m5C/m1A/m7G-related lncRNAs as lncRNAs that were significantly associated with 1 of the 48 m6A/m5C/m1A/m7G genes (*r* > 0.4 and *p* < 0.001). The m6A/m5C/m1A/m7G lncRNA co-expression network generated is shown in Figures 1B,C. Figure 1D describes the associations between the 48 m6A/m5C/m1A/m7G genes and 12 prognostic m6A/m5C/m1A/m7G-related lncRNAs, according to TCGA datasets.

### 3.2 Determination of the m6A/m5C/m1A/m7G-LS

**FIGURE 2:**
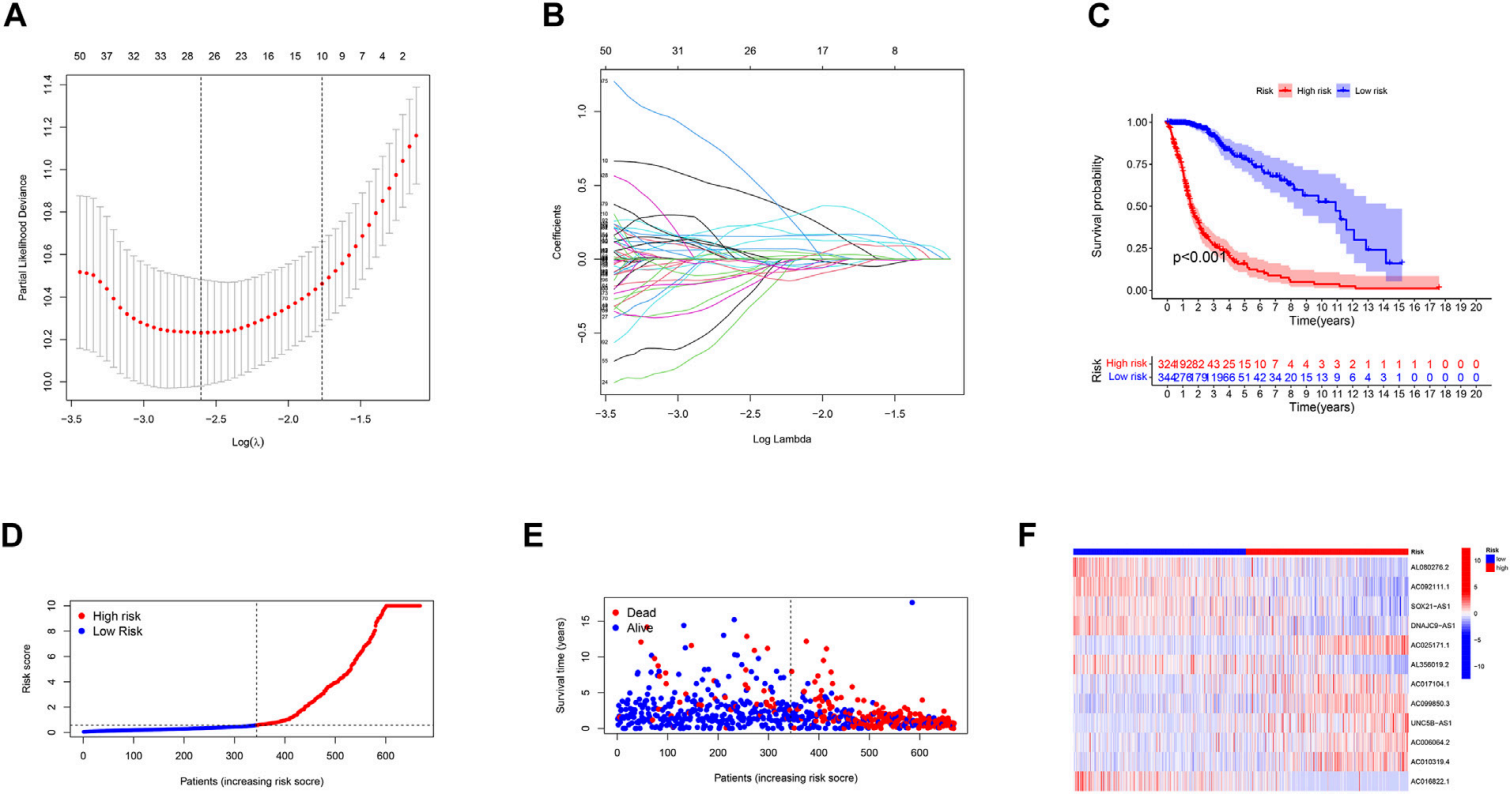
Description of the m6A/m5C/m1A/m7G-LS and evaluation of its prognostic value in TCGA training dataset. **(A)** LASSO analysis of gliomas. **(B)** Determination of the optimal LASSO settings. **(C)** Kaplan–Meier curves of patient OS comparing the high and low m6A/m5C/m1A/m7G-LS groups. **(D)** Distribution of risk scores and patients. **(E)** Dot plot of survival status. **(F)** Heatmap of the expression levels of 12 m6A/m5C/m1A/m7G-related lncRNAs compared between the two groups.

Using univariate Cox regression analysis, we selected m6A/m5C/m1A/m7G-related prognostic lncRNAs from among lncRNAs in the discovery cohort. In TCGA dataset, 488 lncRNAs related to m6A/m5C/m1A/m7G were associated with overall survival (OS). LASSO-penalized Cox is a typical method of multiple regression analysis that not only improves the prediction accuracy of a statistical model but also allows variable selection and regularization. We used LASSO analysis to reduce the overfitting of m6A/m5C/m1A/m7G-LS, resulting in 26 m6A/m5C/m1A/m7G lncRNAs remaining (Figures 2A,B). Finally, 12 lncRNAs associated with m6A/m5C/m1A/m7G (AL080276.2, AC092111.1, SOX21-AS1, DNAJC9-AS1, AC025171.1, AL356019.2, AC017104.1, AC099850.3, UNC5B-AS1, AC006064.2, AC010319.4, and AC016822.1) were considered to be prognosis-related lncRNAs (Table 2).

**TABLE 2:** Multivariate Cox analysis of 12 selected m6A/m5C/m1A/m7G-related lncRNAs.

Readers	Erasers
YTHDC1, YTHDC2, YTHDF1, YTHDF2, YTHDF3, IGF2BP1, IGF2BP2,IGF2BP3, HNRNPA2B1, HNRNPC, HNRNPG, RBMX, FMR1,LRPPRC	FTO,ALKBH5
ALYREF	TET2,YBX1
NOP2, NSUN1, NSUN2, NSUN3, NSUN4, NSUN5,NSUN7, DNMT1, TRDMT1, DNMT3A, DNMT3B	ALKBH1,ALKBH3

Next, glioma samples were stratified into low- and high-risk groups, according to the median value of the prognostic risk level. Kaplan-Meier analysis showed that there was a significant difference between the two groups (*p* < 0.001; Figure 2C). The distributions of risk level, case survival status, and model lncRNA expression levels are shown in Figures 2D–F.

**FIGURE 3:**
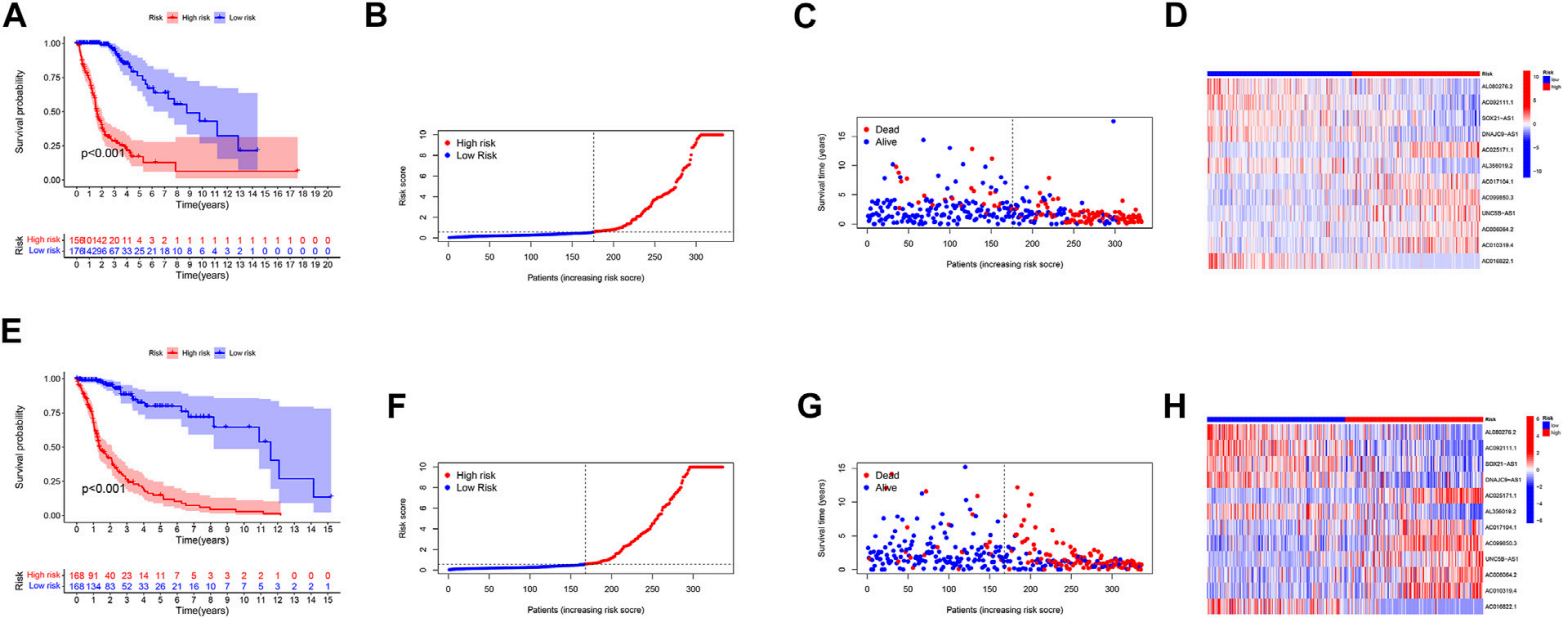
Verification of the m6A/m5C/m1A/m7G-LS in TCGA test and total data sets. **(A,E)** Kaplan–Meier curves of patient OS comparing high and low m6A/m5C/m1A/m7G-LS groups. **(B,F)** Distribution of risk scores and patients. **(C,G)** Dot plot of survival status. **(D,H)** Heatmap comparing the expression levels of 12 m6A/m5C/m1A/m7G-related lncRNAs between the two risk groups.

**FIGURE 4:**
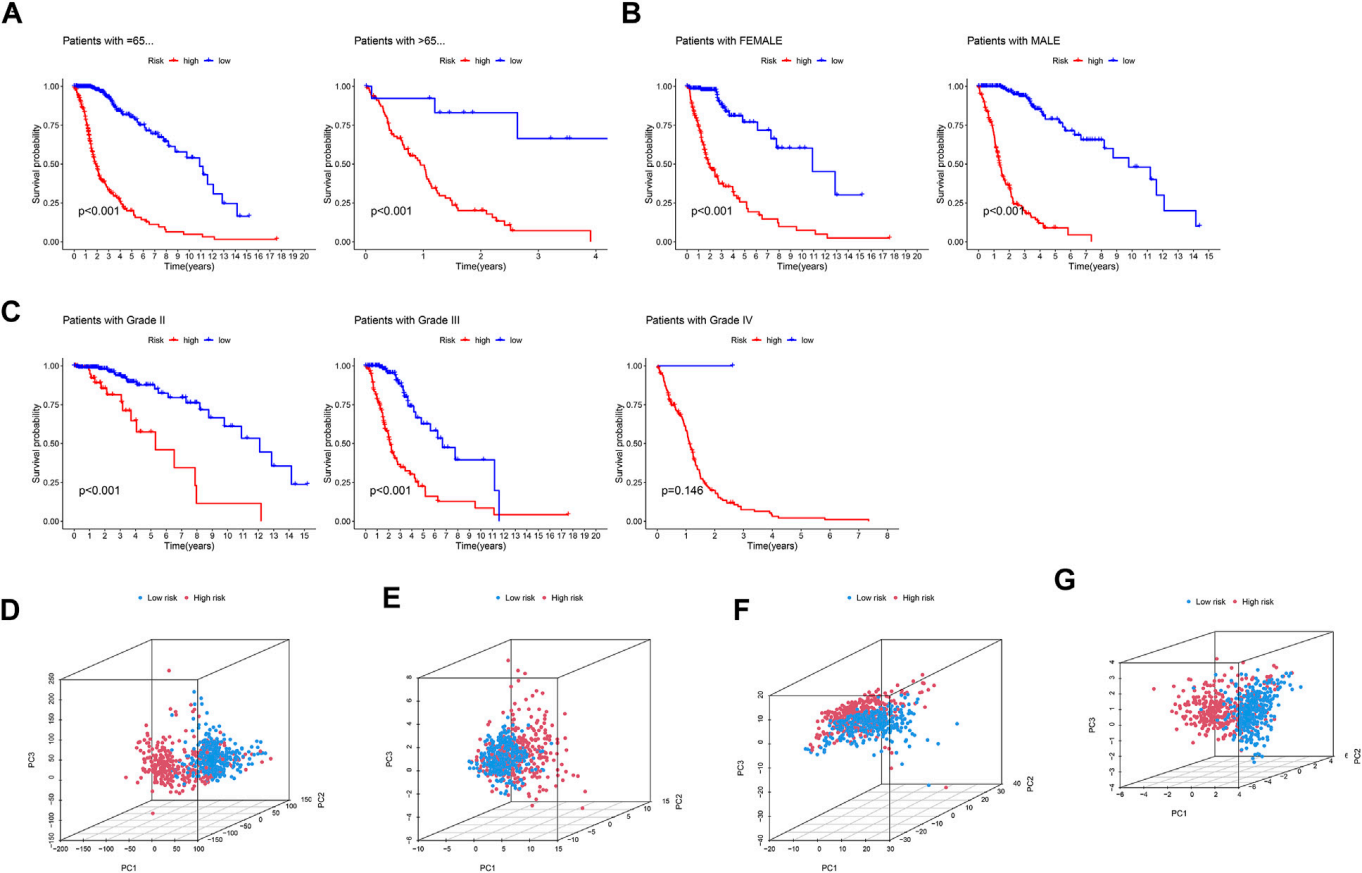
Kaplan–Meier curves of patient OS grouped by **(A)** age, **(B)** sex, and **(C)** tumor grade and compared between the two groups in TCGA total data. PCA comparison between the two groups based on **(D)** entire gene profiles, **(E)** m6A/m5C/m1A/m7G coding genes, **(F)** m6A/m5C/m1A/m7G-related lncRNAs, and **(G)** m6A/m5C/m1A/m7G-LS in TCGA total dataset.

We used standard methods to confirm the reliability of the m6A/m5C/m1A/m7G-LS and verified a similar trend in the validation data (Figure 3). Subsequently, differences in clinical variables between the two stratified groups in the glioma dataset were analyzed. The prognosis of patients in the low m6A/m5C/m1A/m7G-LS group was superior to that in the high m6A/m5C/m1A/m7G-LS group, regardless of sex, age, or subgroup (Figures 4A–C). Further, PCA was used to examine the differences between the two risk groups. The distribution of the two groups was quite scattered (Figures 4D–G), indicating that m6A/m5C/m1A/m7G-LS may differ between them.

**FIGURE 5:**
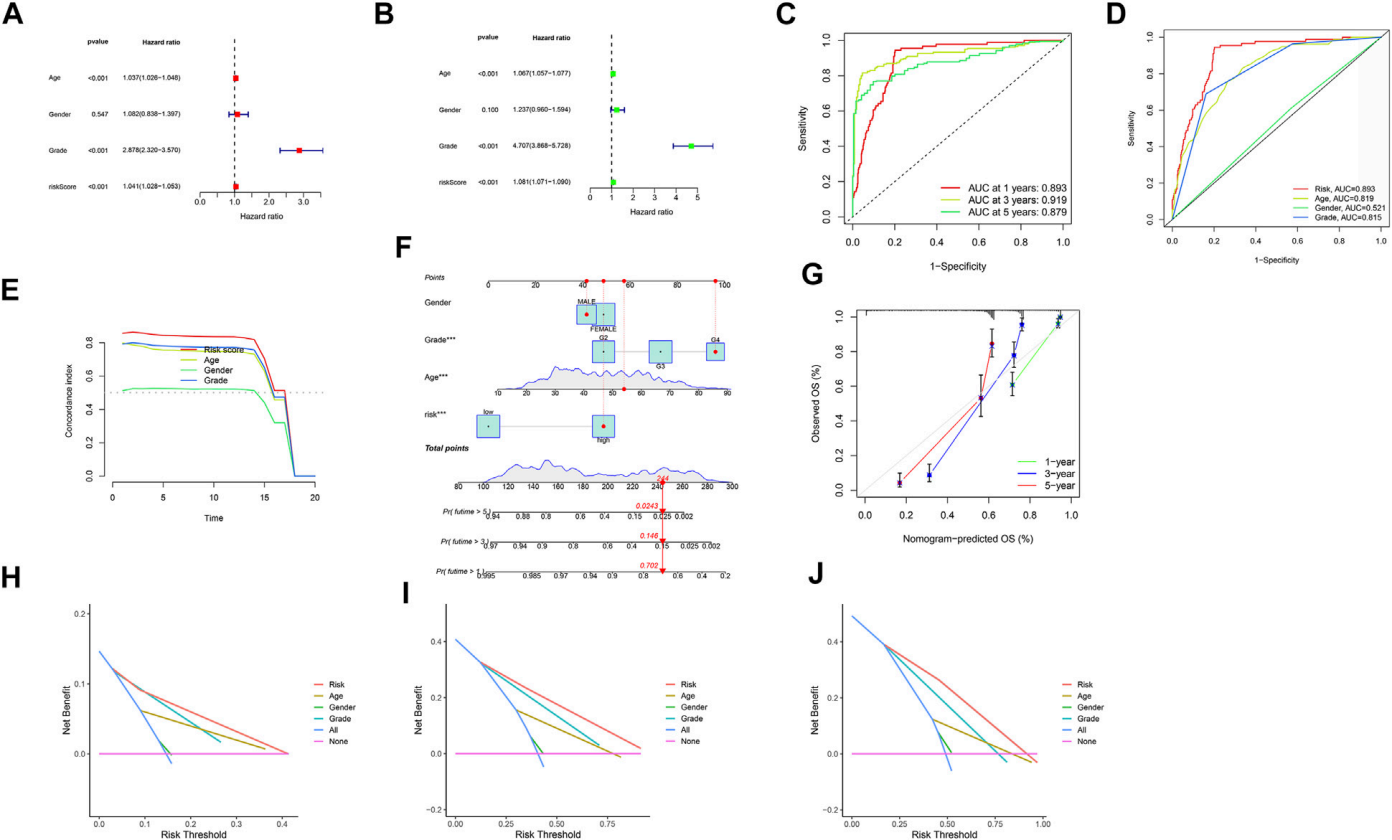
Evaluation of m6A/m5C/m1A/m7G-LS prognostic value and construction of a nomogram model using TCGA total data. **(A,B)** Univariate and multivariate analyses of OS of patients with glioma. **(C)** ROC curves of 1-, 3-, and 5-years OS rates. **(D)** ROC curves of clinical features and risk scores. **(E)** Concordance indices of clinical features and risk scores. **(F)** A nomogram forecasting the 1-, 3-, and 5-years OS rates of patients with glioma. **(G)** Calibration plot of the nomogram model. **(H–J)** DCA of 1-, 3-, and 5-years survival for patients with glioma in TCGA.

Both univariate and multivariate methods revealed the robust independence of our proposed m6A/m5C/m1A/m7G-LS (*p* < 0.001; Figures 5A,B). Area under the receiver operating characteristic (ROC) curve values for 1-, 3-, and 5-years OS rates were all >0.70 (Figure 5C), indicating that the model has a high value for prediction of OS. The area under the ROC curve for risk level was also greater than that for other clinical parameters, indicating that the m6A/m5C/m1A/m7G-LS is relatively reliable for patients with gliomas (Figure 5D). The concordance index of the risk score was higher than that of other clinical indicators, indicating good performance of the m6A/m5C/m1A/m7G-LS (Figure 5E).

Next, we developed a nomogram including risk level and clinical risk characteristics to predict 1-, 3-, and 5-years OS. Using the nomogram, m6A/m5C/m1A/m7G-LS showed greater predictive power than other clinical parameters (Figure 5F). The observed 1-, 3-, and 5-years prediction OS rates showed perfect consistency in correlation analysis (Figure 5G). In DCA, the *y*-axis denotes the net benefits and the *x*-axis indicates the threshold probability. The gray diagonal lines in Figures 5H–J denote the hypotheses that all patients survive for 1, 3, and 5 years, with higher net benefit indicating a superior nomogram.

**FIGURE 6:**
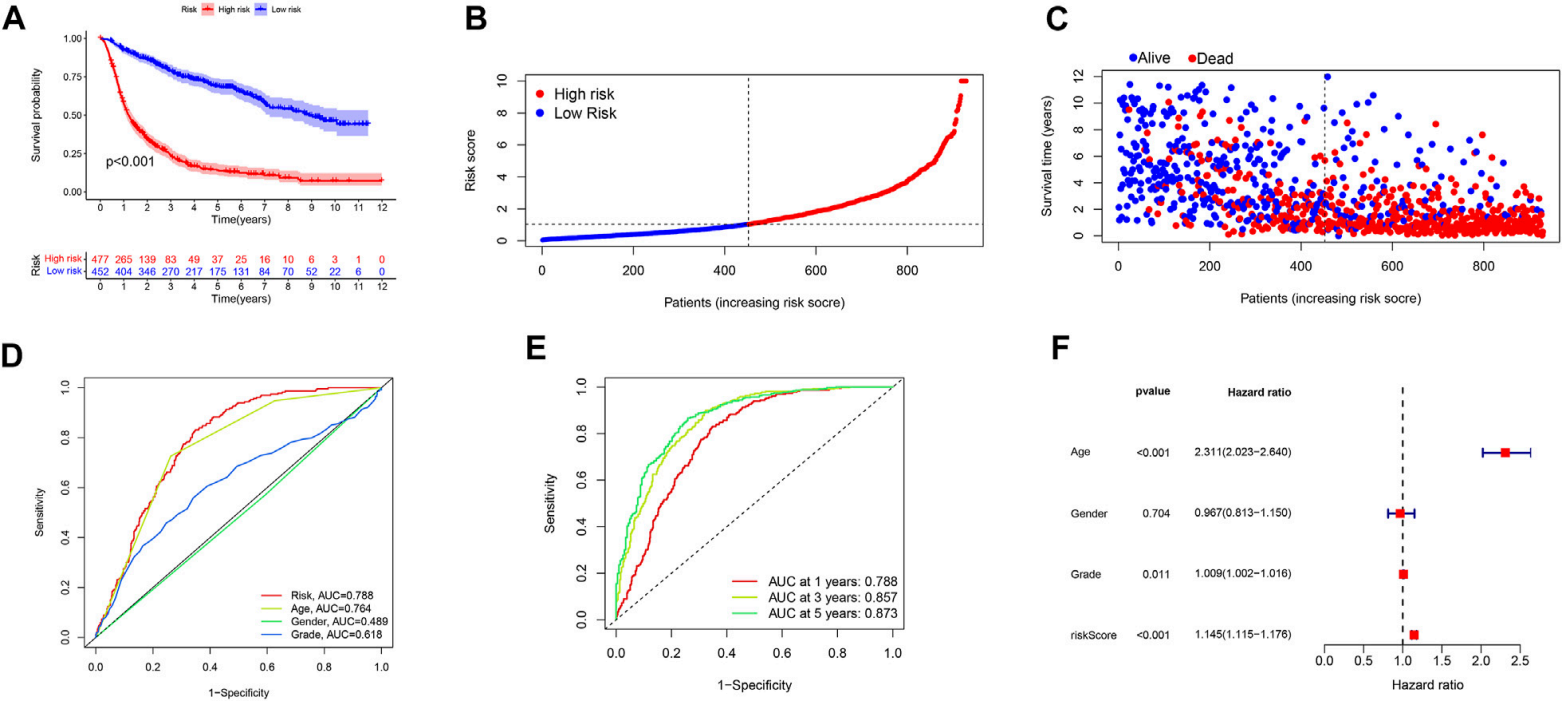
Validation of the signature in the CGGA database. **(A)** Kaplan–Meier curves of patient OS comparing the high and low m6A/m5C/m1A/m7G-LS groups. **(B)** Distribution of risk scores and patients. **(C)** Dot plot of survival status. **(D)** ROC curves of 1-, 3-, and 5-years OS rates. **(E)** ROC curves of clinical features and risk scores. **(F)** Multivariate analyses of OS of patients with glioma.

To investigate the extrapolative accuracy of our signature, we further verified it in CGGA cohorts.The determination of the related-LS was produced via the same formula established in TCGA cohort. Survival analysis, AUC values and multivariate analyses were validated in the CGGA cohort (Figure 6).

### 3.3 Cluster Analysis of m6A/m5C/m1A/m7G-Related LncRNAs in Two Groups of Patients With Glioma

**FIGURE 7:**
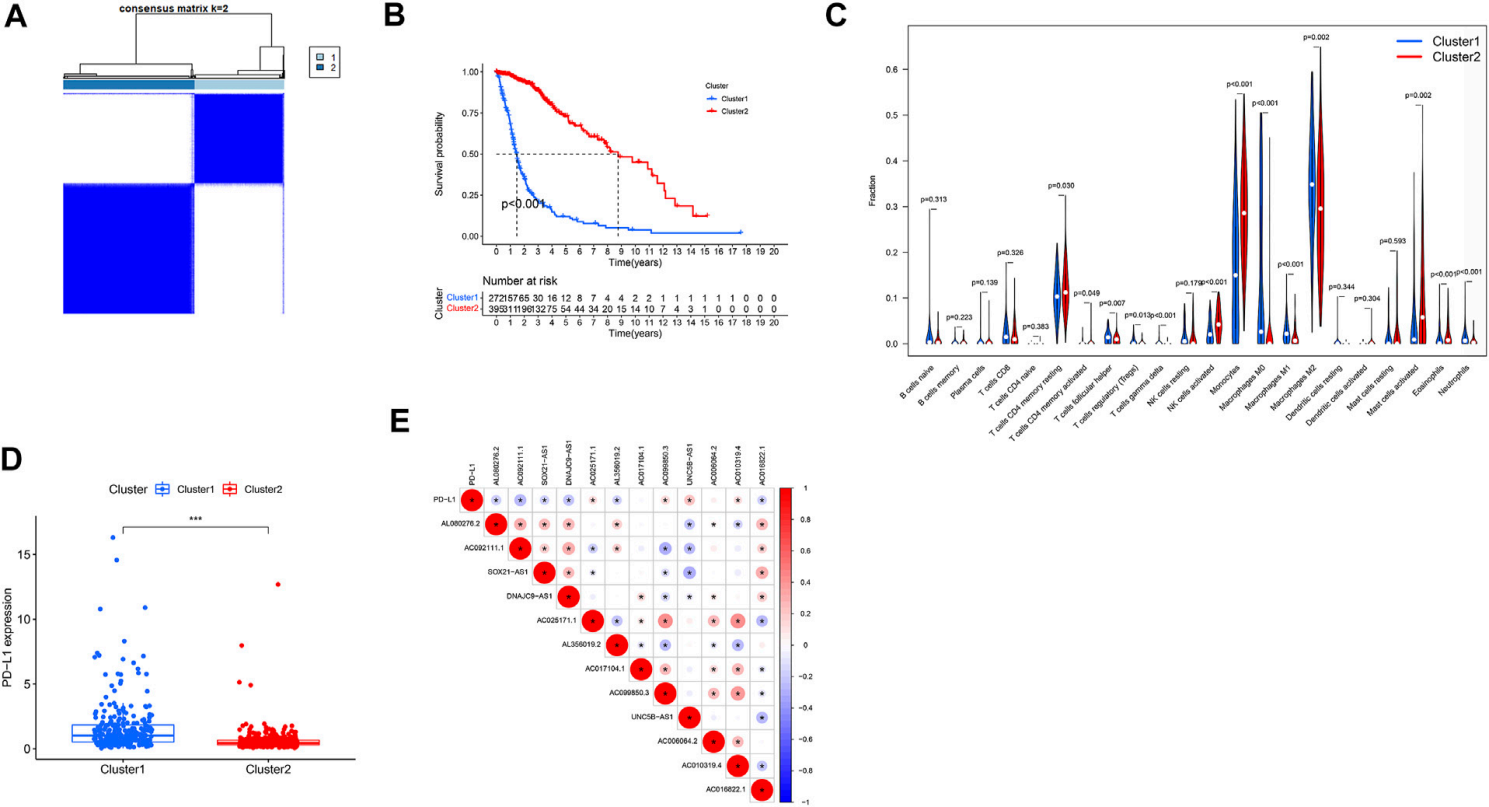
Clinical characteristics and OS of different subgroups of patients with glioma. *p < 0.05, **p < 0.01, and ***p < 0.0001. **(A)** Consensus matrix for optimal k = 2. **(B)** Kaplan–Meier curve of OS time in clusters 1 and 2. **(C)** Violet plot: differential expression of immune cells between clusters 1 and 2 (cluster 1, blue; cluster 2, red). Boxplot **(D)** and correlation coefficient analysis **(E)** of the expression level of PDL1 between clusters 1 and 2.

Tumor samples from TCGA database were divided into two categories by cluster analysis, where the consensus matrix for optimal k = 2 (Figure 7A). Kaplan–Meier survival curve analysis showed that the prognosis of patients in cluster 2 was significantly better than that of patients in cluster 1 (*p* < 0.001; Figure 7B). Analysis of the immune microenvironment of the two subtypes showed that there was significantly less infiltration of resting memory CD4 T cells, activated memory CD4 T cells, activated natural killer (NK) cells, monocytes, activated mast cells, and eosinophils in cluster 1 than cluster 2 (*p* = 0.030, *p* = 0.049, *p* < 0.001, *p* < 0.001, *p* = 0.002, and *p* < 0.001, respectively), while he infiltration of T follicular helper cells, regulatory T cells gamma-delta T cells, M0 macrophages, M1 macrophages, M2 macrophages, and neutrophils was significantly higher in cluster 2 than cluster 1 (*p* = 0.007, *p* = 0.013, *p* < 0.001, *p* < 0.001, *p* < 0.001, *p* = 0.002, and *p* < 0.001, respectively) (Figure 7C). In addition, the expression level of the tumor-suppressor gene, PDL1, was significantly lower in cluster 2 than that in cluster 1 (*p* < 0.001, Figures 7D,E).

### 3.4 Differences in the Immune Microenvironment Between the Low- and High-Risk Groups

**FIGURE 8:**
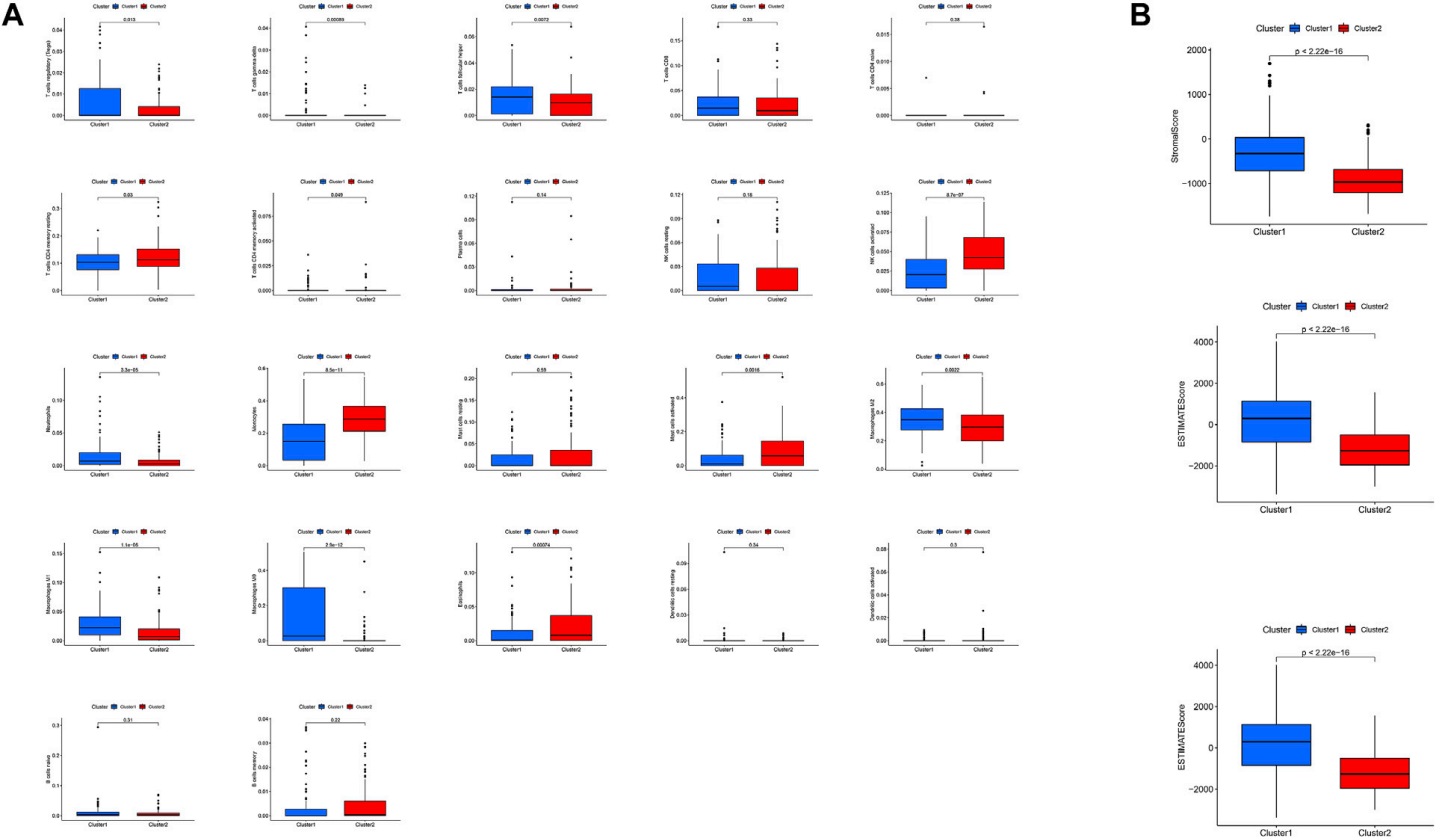
Differences in the immune microenvironment between the high- and low-risk subgroups. **(A)** Violet plot of the infiltration of different immune cells between the high- and low-risk subgroups. **(B)** Analysis of correlations between risk score and immune cell infiltration.

Analysis of the immune microenvironment of the high- and low-risk subtypes showed that, in the first group, the infiltration of CD8 T cells, activated memory CD4 T cells, T follicular helper cells, regulatory T cells, gamma-delta T cells, M0 macrophages, M1 macrophages, M2 macrophages, and neutrophils in the low-risk group was significantly less than that in the high-risk group (*p* < 0.001, *p* = 0.004, *p* = 0.008, *p* = 0.012, *p* < 0.001, *p* < 0.001, *p* < 0.001, and *p* = 0.015, respectively), while resting memory CD4 T cells, activated NK cells, monocytes, activated dendritic cells, activated mast cells, and eosinophils showed significantly greater infiltration in the low-risk group than the high-risk group (*p* = 0.009, *p* < 0.001, *p* < 0.001, *p* = 0.037, *p* < 0.001, and *p* < 0.001, respectively; Figure 8A). Analysis of correlations between risk score and immune cell infiltration also showed that eosinophils, activated mast cells, activated dendritic cells, monocytes, activated NK cells, and resting memory CD4 T cells were negatively correlated with lncRNA grouping. Meanwhile, T follicular helper cells, neutrophils, activated memory CD4 T cells, CD8 T cells, regulatory T cells, M0 macrophages, M1 macrophages, and M2 macrophages were positively correlated with risk score (*p* < 0.05; Figure 8B).

### 3.5 Evaluation of Immunotherapy

**FIGURE 9:**
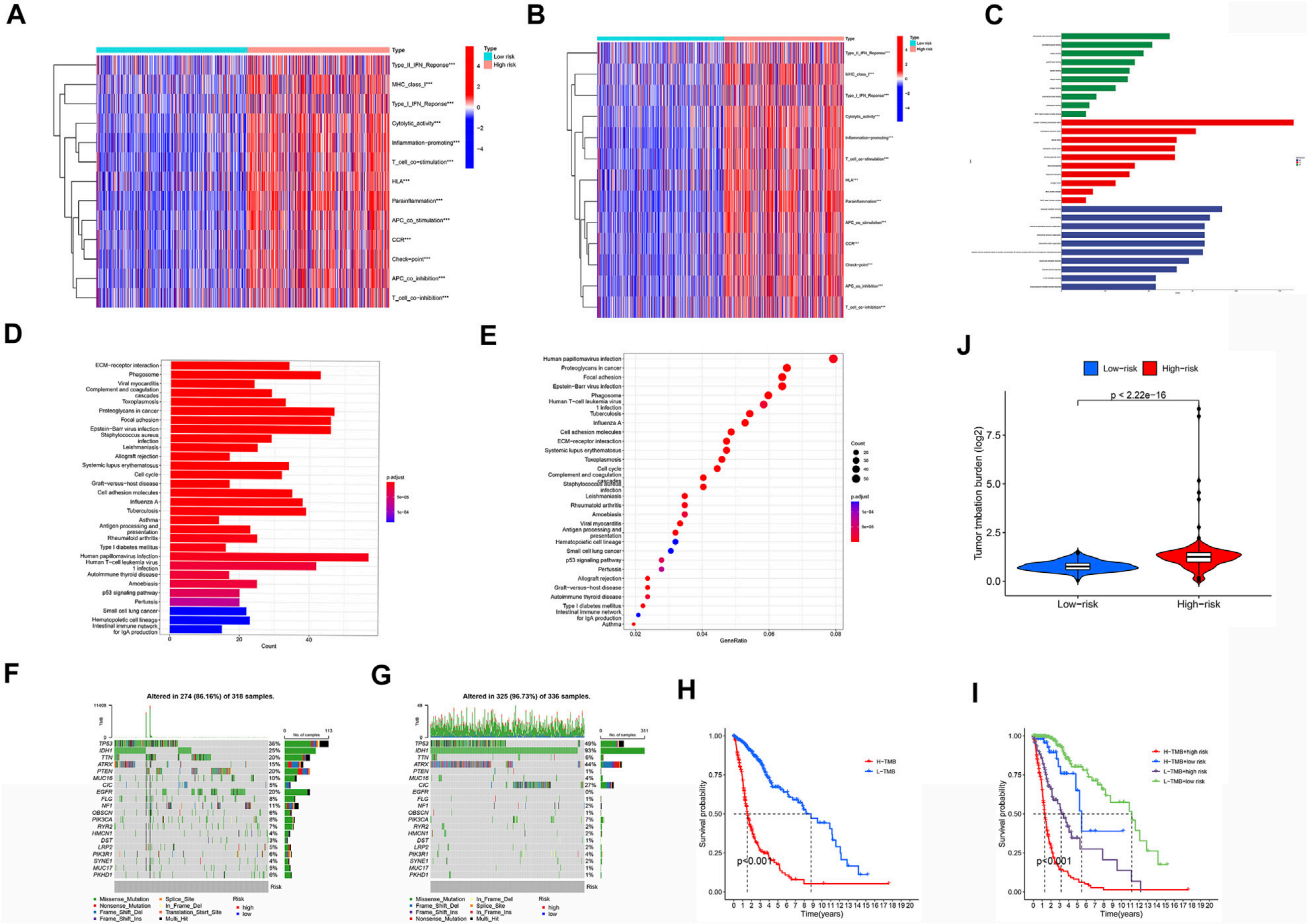
Immune status, enriched pathways, and immune activity in glioma samples. **(A)** Comparison of the immune status landscapes of the two groups. **(B,C)** GO enrichment analysis. **(D,E)** KEGG enrichment analysis. **(F)** Differences in TIDE results between the two groups. **(G,H)** Waterfall plot of mutation values in the high **(G)** and low **(H)** m6a/m5C/m1A/m7G-LS groups. **(I)** Comparison of TMB between the two groups. **(J)** Kaplan-Meier analysis based on TMB. **(K)** Kaplan-Meier analysis combining TMB and the risk signature. *p < 0.05, **p < 0.01, and ***p < 0.001.

Based on the function of the m6A/m5C/m1A/m7G-LS, we also studied immune status, enriched pathways, and immune activity in glioma samples. The expression of immunomarkers differed significantly between the low and high m6A/m5C/m1A/m7G-LS groups (Figure 9A). We used GO analysis to study possible molecular processes associated with m6A/m5C/m1A/m7G-LS, and the results suggested involvement in several immune-related biological processes (Figure 9B,C). KEGG analysis was performed to investigate possible pathways enriched for m6A/m5C/m1A/m7G-LS, and suggested the involvement of several immune-related pathway processes (Figure 9D,E).

Next, we examined the relationship between m6A/m5C/m1A/m7G-LS and immunotherapy biomarkers. Predictably, the high m6A/m5C/m1A/m7G-LS group was more likely to respond to immunotherapy than the low m6A/m5C/m1A/m7G-LS group, suggesting that the m6A/m5C/m1A/m7G-LS-based classifier score may be useful for predicting the results of TIDE analysis (Figure 9F).

Tumor mutation data were evaluated and summarized using the R package “Maftools”. Variation effect predictors were used to stratify mutations. The top 20 genes with the greatest numbers of mutations in the two groups are presented in Figures 8G,H. TMB scores were then generated using TGCA somatic mutation data, and m6A/m5C/m1A/m7G-LS was found to be strongly associated with TMB (Figure 9I) . Further, high TMB was associated with poor OS (*p* < 0.001;Figure 9J). Next, we investigated whether the combination of m6A/m5C/m1A/m7G and TMB could be a more powerful prognostic biomarker. All samples were divided into four groups based on risk factors and TMB, as follows: high TMB + high m6A/m5C/m1A/m7G-LS, low TMB + low m6A/m5C/m1A/m7G-LS, low TMB + high m6A/m5C/m1A/m7G-LS, and high TMB + low m5C m6A/m5C/m1A/m7G-LS. As shown in Figure 8K, there were significant differences between these groups (*p* < 0.001), with the highest OS recorded in the low TMB + low m6A/m5C/m1A/m7G-LS group. These results clearly demonstrate that m6A/m5C/m1A/m7G-LS is associated with tumor invasiveness.

### 3.6 Identification of a New Compound Targeting m5C-sLS

**FIGURE 10:**
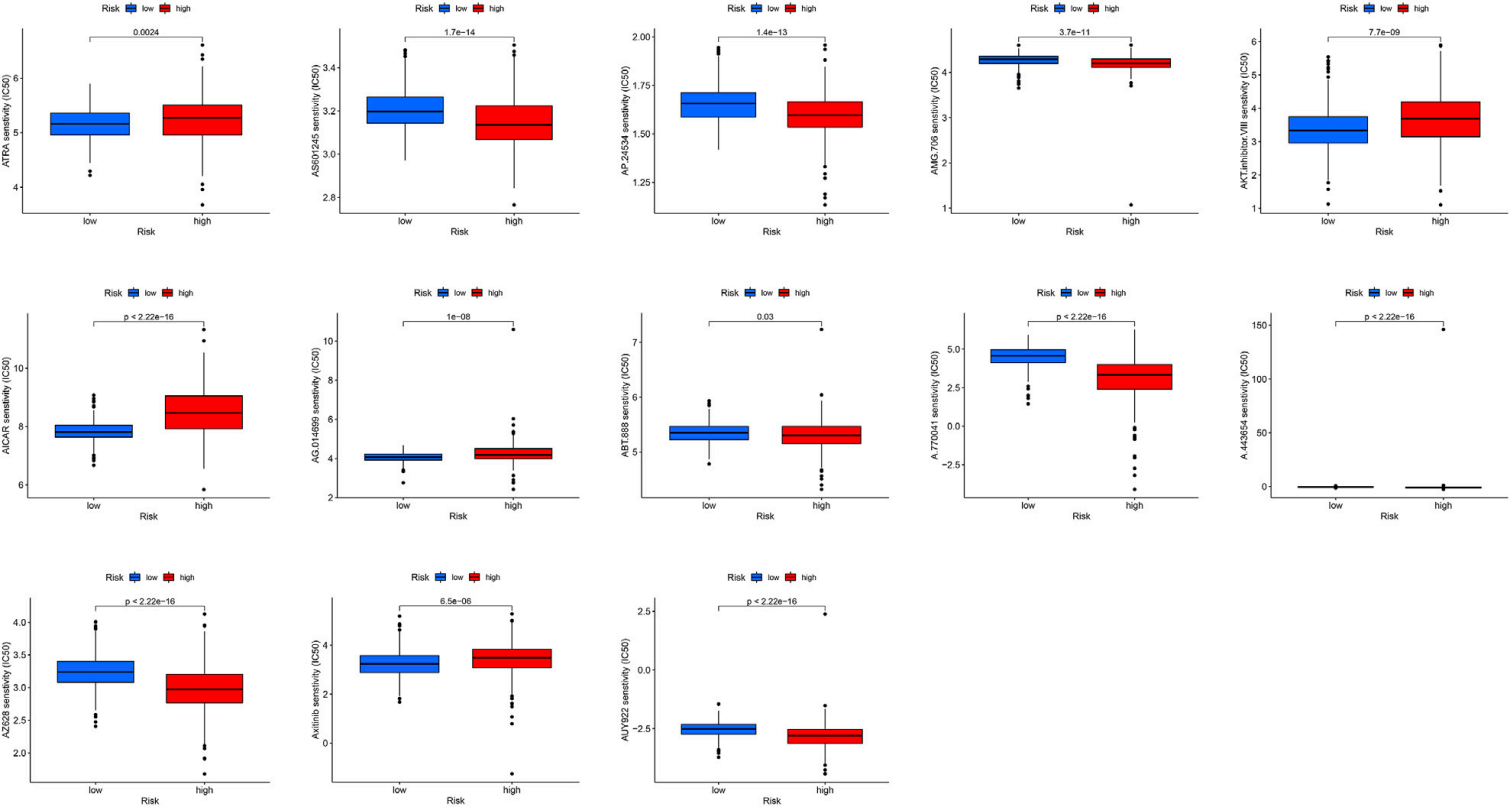
Boxplot of the results of drug sensitivity analysis in the high- and low-risk subgroups.

We used the pRRophetic algorithm to assess which drugs may be effective for glioma patients by collating IC_50_ values for each sample from the Genomics of Drug Sensitivity in Cancer database. Thirteen compounds were screened based on significant differences in predicted IC_50_ values between the two groups, and the high LS group was more sensitive to most compounds. As illustrated in Figure 10, the 13 drugs that warrant further study in glioma include an Akt modulator (a-443654), an Src family selective Lck inhibitor (a-770041), a poly (adenosine diphosphate–ribose) polymerase 1 inhibitor (ABT-888), veliparib, rucaparib (AG.014699), 5-aminoimidazole-4-carboxamide ribonucleoside (AICAR), Akt inhibitor VIII, an oral multikinase inhibitor (AMG-706), a third-generation kinase inhibitor (AP.24534), a c-Jun NH2-terminal kinase inhibitor (AS601245), the vitamin A metabolite, all-trans retinoic acid (ATRA), a heat shock protein 90 inhibitor (AUY922), AXITINIBJ, and a BRAF kinase inhibitor (AZ628). Analysis of drug sensitivity showed that patients in the low-risk group were predicted to be more sensitive a-770041, amg-706, ABT-888, ap-24534, as601245, auy922, and az628 than those in the high-risk group, while patients in the high-risk group were predicted to be more sensitive to axitinib, a-443654, ag-014699, AICAR, Akt inhibitor VIII, and ATRA than those in the low-risk group.

## 4 Discussion

Dynamic RNA methylation and modification events, such as m6A, m5C, m1A, and m7G, are involved in tumor progression, migration, invasion, and epithelial–mesenchymal transition of cancer cells, both *in vitro* and *in vivo* (Michalak et al., 2019). Modification events can also serve as prognostic markers and play indispensable roles in various tumors (Han et al., 2019; Müller et al., 2019; Zhang C. et al., 2020; Liu T. et al., 2020; Guo et al., 2020). There is evidence for an interaction between lncRNA and RNA methylation in tumors. For example, loss of the lncRNA, THOR, inhibits the proliferation, migration, and invasion of cancer cells *in vitro* and *in vivo,* while the m6A readers, YTHDF1 and YTHDF2, can regulate THOR, thereby inhibiting the occurrence and development of tumors *in vivo* and *in vitro* (Liu H. et al., 2020). In addition, Linc00022 can be inhibited by the m6A reader, YTHDF2, to influence the invasion and proliferation of esophageal squamous cell carcinoma (Cui et al., 2021). Moreover, m5C can modify the lncRNA, H19, to recruit oncoproteins and promote tumor proliferation and invasion (Sun et al., 2020). LINC00857 expression is also mediated by m6A, and can promote the development of pancreatic cancer (Meng et al., 2021). Conversely, lncRNA can also affect the function of genes related to RNA methylation modification. For example, knockdown of Nutm2a-as1 can regulate Mettl3 to inhibit lung cancer progression (Wang J. et al., 2021). Song et al. used a systems approach to integrate gene expression and patient survival data with protein interaction networks in discrete windows of tumor proliferative biology and demonstrated that, in combination, these networks may form the basis of highly accurate prognostic classification models, with potential clinical utility for guiding therapeutic options for patients (Song et al., 2015). Su et al. also used a multi-omics approach to characterize brain metastasis, providing an opportunity to identify clinically impactful biomarkers and associated prognostic subtypes, thereby generating an integrative understanding of disease (Su et al., 2020). Overall, the evidence presented above suggests that RNA methylation and lncRNA act together to influence the tumorigenesis of various cancers; however, whether RNA methylation is involved in glioma development by its influence on lncRNA has yet to be fully elucidated.

At present, the signatures used to predict prognosis of patients with glioma are sub-optimal. Chen et al. (2022) constructed prognostic signatures of N6-methyladenosine-related lncRNAs in gliomas, demonstrating the feasibility of applying prognostic signatures for patients with glioma. Maimaiti et al. (2022) analyzed the relationships between clinical outcomes and methylation-related lncRNAs in LGG and the tumor microenvironment, and found that gliomas are characterized by the tumor microenvironment. Liu X et al. (2022) also used m5C-related lncRNAs for prognostic prediction and immune response signature analysis, while Wang et al. demonstrated the prognostic value and immune landscape of lncRNAs associated with m6A/m5C/m1A in squamous cell carcinoma of the head and neck. We wished to improve on these prognostic models in gliomas; therefore, we integrated data from m6A/m5C/m1A/m7G-related genes, constructed a prognostic signature comprising relevant lncRNAs, determined its prognostic value and relationship with the immune landscape, and conducted immune infiltration and drug sensitivity analyses. Such prognostic features can be applied as independent factors for superior prediction of the prognosis of patients with glioma, opening new possibilities for immunotherapy approaches targeting glioma in the future. Therefore, in this study, we sought to investigate whether relevant lncRNAs are associated with immunotherapy and involved in the occurrence and development of gliomas.

Data from five normal and 698 tumor samples were downloaded from TCGA database to search for m6A/m5C/m1A/m7G-related genes. We first selected 488 m6A/m5C/m1A/m7G-related lncRNAs from TCGA dataset and constructed lncRNA pairs using a 0-or-1 matrix. Finally, A 12-factor m6A/m5C/m1A/m7G-LS (AL080276.2, AC092111.1, SOX21-AS1, DNAJC9-AS1, AC025171.1, AL356019.2, AC017104.1, AC099850.3, UNC5B-AS1, AC006064.2, AC010319.4, and AC016822.1) was developed based on these prognostic lncRNA pairs using a 1000-iteration LASSO regression model, as proposed by Sveen et al. (2012). Prognostic gene pairs were selected based on their frequency, rather than on intersections, in the 1000 random stimulation iterations, for a more accurate prediction. The m6A/m5C/m1A/m7G-LS was shown to have predictive significance and was then used to identify prognostic characteristics. The lncRNA, AC099850.3, promotes hepatocellular carcinoma proliferation and invasion through the PRR11/PI3K/AKT axis and is associated with patient prognosis (Zhong et al., 2022), indicating that it is an important prognostic indicator. The lncRNA, SOX21-AS1, is hypomethylated in cervical cancer, can serve as a new biomarker for the diagnosis of cervical squamous cell carcinoma, and is a potential therapeutic target. Moreover, SOX21-AS1 is correlated with clinical stage of nephroblastoma and regulates cell proliferation, which is a likely prognostic marker of nephroblastoma (Zhang et al., 2019; Du et al., 2021). UNC5B-AS1 is highly expressed in various tumors and promotes tumor proliferation, migration, and invasion (Wang et al., 2019; Wang H. et al., 2020; Tan et al., 2020). Notably, the significance of the remaining lncRNAs has not been clearly elucidated to date, offering a future research direction. We plan to conduct subsequent studies on these genes to determine their relationships with RNA methylation and further assess their associations with glioma invasion, migration, and proliferation. Subsequently, glioma patients were divided into high and low m6A/m5C/m1A/m7G-LS groups, according to median score, and the high-risk group was found to have inferior clinical prognosis. Similar results were found on analyses of subgroups disaggregated by sex, age, and tumor stage. Further, PCA confirmed the grouping ability of the m6A/m5C/m1A/m7G-LS. Multivariate Cox analysis showed that this model may be an independent risk factor for OS in patients with glioma. We also created a bar chart illustrating the perfect agreement between observed and predicted 1-, 3-, and 5-years OS rates. Therefore, the m6A/m5C/m1A/m7G-LS model established here may assist in discovery of new biomarkers for future application.We further verified the extrapolative accuracy of our signature in CGGA cohorts. Survival analysis, AUC values and multivariate analyses were validated in the CGGA cohort. it could be suggested that the 13-gene risk score can independently evaluate the survival of patients with gliomas.

Tumor samples were subsequently divided into clusters 1 and 2, according to their expression levels of the 12 selected lncRNAs. The survival rate of patients with cluster 2 tumors was significantly better than that of those with cluster 1 cancers. We further studied the tumor microenvironment of the samples based on these two clusters and risk score classifications and found that there were significant differences in the infiltrations of different immune cells between clusters 1 and 2 and between the high- and low-risk subgroups, revealing the characteristics of the tumor immune microenvironment among these different subgroups. At present, few patients with glioma benefit from immunotherapy. Therefore, it is necessary to identify new biomarkers to optimize treatment strategies. Checkpoint inhibitors, such as PDL1 inhibitors, are used for treatment of numerous cancers (Gato-Cañas et al., 2017), and there are many ongoing trials focusing on the identification of novel biomarkers in gliomas (Saadatpour et al., 2016). In addition, PDL1 may be associated with various lncRNAs, and the lncRNA, PSMB8-AS1, promotes pancreatic cancer progression by regulating the miR-382-3p/STAT1/PDL1 axis (Zhang H. et al., 2020). PDL1 and JAK2 transcripts are negatively regulated by binding to the nuclear ribonucleic protein, HNRNPH1. Primary INCR1 transcripts bind HNRNPH1 to block its inhibition of PDL1 and JAK2, enabling their expression (Mineo et al., 2020). We explored the expression of PDL1 in the two tumor sample clusters and the relationship between PDL1 and the 12 selected lncRNAs, demonstrating that PDL1 expression may be associated with these lncRNAs. These findings suggest the possibility of selecting differential immune checkpoint genes as therapeutic targets for patients with gliomas.

In addition, we analyzed combined TMB and risk score models and showed that patients with low mutation loads had better prognosis than those with high mutation loads, while patients with low mutation loads in the low-risk subgroup had the best prognosis and those with high mutation loads in the high-risk subgroup had the worst prognosis, clearly demonstrating the importance of TMB in predicting prognosis. The TIDE algorithm, which simulates tumor immune-evasion pathways, was used to predict cancer therapy outcomes in response to blocking of immune checkpoints (Jiang et al., 2018). Our results showed that patients with glioma with high risk scores were predicted to respond better to immunotherapy. TMB refers to somatic coding mutations related to the formation of anti-tumor neo-antigens (Gubin et al., 2015), where higher TMB values are associated with stronger killing effects of T cells stimulated by PD1, and superior clinical effects (Allgäuer et al., 2018). TMB was greater in the high m6A/m5C/m1A/m7G-LS group than that in the low m6A/m5C/m1A/m7G-LS group, suggesting that immunotherapy would be more effective in the high m6A/m5C/m1A/m7G-LS group. In addition, the combination of TMB with lncRNAs associated with m6A/m5C/m1A/m7G yielded good predictive results. Therefore, this study contributes to our understanding of the molecular biological role of m6A/m5C/m1A/m7G-related lncRNAs in gliomas. Considering the therapeutic potential of these 12 lncRNAs, we analyzed their sensitivity to different small-molecule drugs. Sensitivity to A.770041, AMG.706, ABT.888, AP.24534, AS601245, AUY922, and AZ628 was higher in the low-risk group than the high-risk group, while sensitivity to axitinib, A.443654, AG.014699, AICAR, Akt inhibitor VIII, and ATRA was higher in the high-risk group than the low-risk group. These drugs are commonly used in the clinical treatment of gliomas, and our results support their therapeutic value in this context (Djuzenova et al., 2012; Yuan et al., 2018; Wang et al., 2021c; Milton et al., 2021). In addition, these findings suggest the prospect of targeting lncRNAs in therapy for patients with gliomas.

This study has some limitations. First, the data used in the study came from TCGA database, and validation in a separate patient cohort was lacking. Further, the predictive value of m6A/m5C/m1A/m7G-LS for clinical application requires additional evaluation.

## 5 Conclusion

The poor prognosis of patients with gliomas affects the health of tens of millions of people each year, and many researchers are focused on trying to improve the prospects for these individuals. RNA methylation is established as involved in cancer progression. Several studies have used lncRNAs to ascertain prognostic markers, to identify new targets for cancer diagnosis and treatment. Our findings illustrate how lncRNAs associated with m6A/m5C/m1A/m7G are associated with glioma prognosis, the immune microenvironment, TMB, and drug sensitivity. Such prognostic features can be better applied as independent factors for predicting the prognosis of patients with glioma, opening new possibilities for immunotherapy approaches targeting glioma in the future.

## Data Availability

The original contributions presented in the study are included in the article/Supplementary Materials, further inquiries can be directed to the corresponding author.
